# SARS-associated Coronavirus Transmitted from Human to Pig

**DOI:** 10.3201/eid1103.040824

**Published:** 2005-03

**Authors:** Weijun Chen, Minghua Yan, Ling Yang, Boliang Ding, Bo He, Yingzhen Wang, Xiuli Liu, Chenhui Liu, Hui Zhu, Bo You, Shengyong Huang, Jiangguo Zhang, Feng Mu, Zhao Xiang, Xiaoli Feng, Jie Wen, Jianqiu Fang, Jun Yu, Huanming Yang, Jian Wang

**Affiliations:** * Chinese Academy of Sciences, Beijing, China; † Beijing BGI-GBI Biotech Co., Ltd, Beijing, China; ‡ Tianjin Institute of Animal Husbandry and Veterinary Science, Tianjin, China; § BGI Hangzhou Bio-Environment Technology Co., Ltd, Hangzhou, China; 1 W. Chen, M. Yan, and L. Yang contributed equally to this article.

## Abstract

coronavirus (SARS-CoV) was isolated from a pig during a survey for possible routes of viral transmission after a SARS epidemic. Sequence and epidemiology analyses suggested that the pig was infected by a SARS-CoV of human origin.

Severe acute respiratory syndrome (SARS) was first identified in Guangdong Province, China, in November 2002 (*1*). A novel coronavirus, SARS-CoV, was identified as the pathogen; several possible origins of the coronavirus were suggested from wild animal reservoirs, such as Himalayan palm civets and raccoon dogs (*2*–*8*). The virus infects many other wild and domesticated animals, such as *Mustela furo*, *Felis domesticus*, and *Nyctereutes procyonoides* (*9*,*10*), but infection of domesticated pigs has not been previously reported.

## The Study

We surveyed 6 major domestic animal species that are in close contact with humans and could be infected by SARS-CoV if transmission were possible. The survey was conducted in a suburban area and its extended farming villages, Xiqing County of Tianjin, China, where a SARS outbreak occurred in late spring of 2003. Animal samples, blood and fecal swab specimens, for antibody and RNA detection were collected from the sites and transported on ice to a biosafety level 3 laboratory within 24 hours. We used 2 types of assays for the initial viral screen, immunologic assays to identify antibodies and reverse transcription–polymerase chain reaction (RT-PCR) to detect the viral genome. The immunoassays were carried out by the double-antigen sandwich method with a recombinant N protein and a partial S protein of SARS-CoV, and results were confirmed by Western blot (*11*). RT-PCR with virus-specific primers was used to detect viral genome RNA, which was extracted from blood samples with a QIAamp RNA Blood Mini Kit (Qiagen, Hilden, Germany) and from fecal swabs with Trizol Reagent (Invitrogen, Carlsbad, CA, USA).. Total RNA was then reverse transcribed with random hexamers, and cDNA was amplified with a nested PCR method (*12*). We also isolated viruses from Vero E6 cultures, performed a cross-neutralization test, and sequenced the viral genome (*13*).

**Figure 1:**
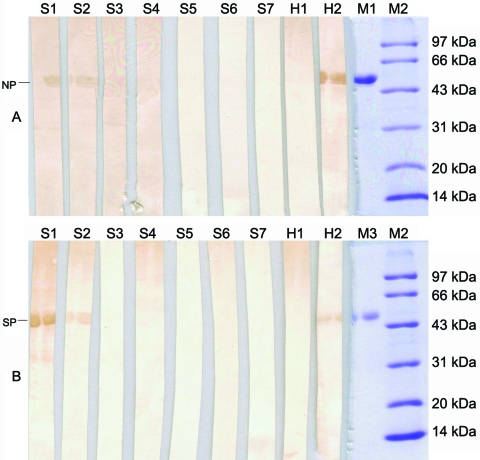
Detection of antibodies against severe acute respiratory syndrome (SARS)–associated coronavirus recombinant proteins in animal sera by Western blotting. Recombinant nuleocapsid protein in panel A (NP, 54 kilodaltons [kDa]) and partial spike protein in panel B (SP, 57 kDa) were used as antigens. Goat anti-swine immunoglobulin G horseradish peroxidase was used as a secondary antibody. Serum samples from a convalescent SARS patient and healthy persons were used as positive and negative controls, respectively. Swine (S1 to S8) and human (H1 and H2) samples are sera collected during the survey. M1, M2, and M3 are purified NP, SP, and molecular weight markers, respectively. Positive bands at the corresponding molecular weight of the 2 proteins are indicated with arrows.

Of 242 animals surveyed, we identified 2 antibody-positive samples from 2 pigs; test results for the other 240 animals were negative (Table 1 and Figure 1). Of 93 blood specimens and 15 fecal swabs on which we performed RT-PCR, 1 of the same 2 pigs tested positive. We subsequently obtained 2 viral isolates from its blood and fecal samples, designated TJB and TJF, respectively. We also performed follow-up studies for 4 weeks on the infected pig until its blood tested negative with our RT-PCR assay. The animal later died giving birth. We also tested serum samples from the swineherd on the farm and a few persons who may have had contact with the swineherd. All were negative by the tests that we conducted.

**Table 1:** Animal surveys for antibodies to SARS-CoV and viral RNA*

Animals	Antibodies in serum samples				RT-PCR				Virus isolation			
	ELISA		WB		Blood		Fecal swab		Blood		Fecal swab	
	N	P	N	P	N	P	N	P	N	P	N	P
Pigs	108	2	5	2	14	1	14	1	6	1	6	1
Cattle	60	0	0	0	22	0	0	0	0	0	0	0
Dogs	20	0	0	0	14	0	0	0	0	0	0	0
Cats	11	0	0	0	11	0	0	0	0	0	0	0
Chickens	11	0	0	0	11	0	0	0	0	0	0	0
Ducks	30	0	0	0	20	0	0	0	0	0	0	0

*Numbers in table refer to number of animals tested with the assays. SARS-CoV, severe acute respiratory syndrome–associated coronavirus; RT-PCR, reverse transcriptase–polymerase chain reaction; ELISA, enzyme-linked immunosorbent assay; WB, Western blot; N, negative; P, positive.

**Figure 2:**
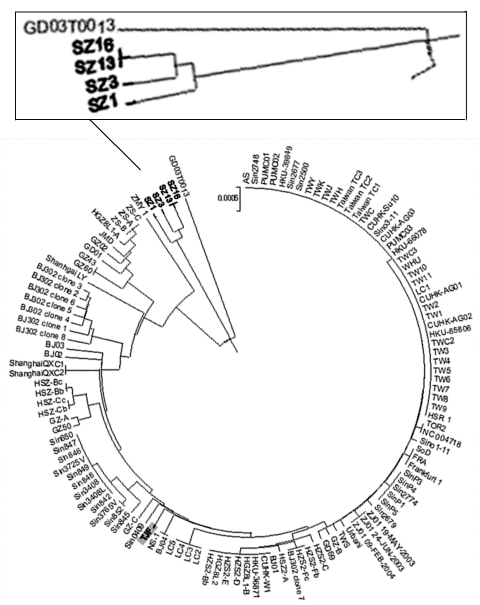
Phylogenetic analysis of severe acute respiratory syndrome–associated coronavirus S-gene. Nucleotide sequences of S genes (from 21491 to 25258 and 3768 bp in length) were compared. The result was displayed with MEGA-2 program and based on 125 complete S-gene sequences from GenBank.

Using a viral isolate, TJF, we conducted cross-neutralization experiments with antisera and an early viral isolate, BJ01 (*8*), to prove their equivalent virulence (Table 2). We then sequenced TJF completely (GenBank accession no. AY654624) and compared its sequence to that of BJ01. Eighteen nucleotide (nt) substitutions are between the TJF and BJ01 sequences, and 4 of them are nonsynonymous over the entire length (29,708 bp). Two pieces of evidence strongly suggested a human origin for the TJF strain. First, it is only distantly related to SZ16, which was isolated from Himalayan palm civets of southern China, in which 64 substitutions over a length of 29,731 bp were found, 3.6 times more than were identified between TJF and BJ01. Second, a sequence signature (a 29-nt insertion [246 nt upstream of the N gene, from residues 27869 to 27897]) found only in an early isolate, GD01 (from Guangdong Province), but absent from all the SARS-CoV isolates so far discovered, is also absent in the TJF sequence (*14*). This sequence has been found in all coronavirus isolates of animal origin except from the pig identified in this study. Therefore, direct viral transmission of SARS-CoV from a human host to the pig bearing TJF is most likely. To further elucidate our point, we constructed a phylogenetic tree based on S-gene sequences; it shows that TJF is more closely related to human SARS-CoV isolates than to animal coronaviruses (Figure 2).

**Table 2:** Cross-neutralization tests for severe acute respiratory syndrome (SARS)–associated coronavirus*

Virus strains	Sera (ND_50_)					
	S1	S2	S3	H1	H2	H3
TJF	1:160	1:640	<1:10	1:1,280	1:640–1,280	<1:10
BJ01	1:160–320	1:640	<1:10	1:640–1,280	1:320–640	<1:10

*S1 and S2 were sera from swine that were positive by enzyme-linked immunosorbent assay (ELISA). H1 and H2 were sera from SARS patients. H3 and S3 were controls from sera of a normal human and an ELISA-negative pig, respectively. ND_50_, 50% neutralization dose.

## Conclusions

We have shown that human SARS-CoV can infect domesticated mammals, in particular, the pig. The direct source of SARS-CoV transmission to the identified infected pigs was most likely virus-contaminated animal feed because the farm where the infected pig was identified is rather remote, >1 km from the nearest village. The only person routinely in close contact with the animals is the swineherd, whose serum samples were negative for SARS-CoV on all tests. Swineherds in rural areas often obtain leftovers from restaurants in the cities for use as hogwash (without thoroughly fermenting it). Thus, even if no direct evidence for human-to-swine SARS-CoV transmission exists, a strong warning should be issued to prevent such a practice, or regulatory procedures should be instituted to block this route of disease propagation (*15*). Whether or not other domesticated (such as dogs and cats) and wild animals that are common in and around human settlements can easily contract and pass on SARS-CoV remains to be seen in future studies. Intensive surveillance and investigations on animals, especially during and after an outbreak of SARS, will lead to a better understanding and ability to control this disease’s natural animal reservoirs and to prevent interspecies transmission events.
